# Supplementary material for: Utility of TEMPS-A in differentiation between major depressive disorder, bipolar I disorder, and bipolar II disorder
Source: PLoS One. 2020 May 22;15(5):e0232459. doi: 10.1371/journal.pone.0232459 (PMC7244116; doi:10.1371/journal.pone.0232459)
Supplement: S1 Table — (DOCX) [file pone.0232459.s001.docx]

| **Table S1. Multivariate logistic regression analysis of the diagnosis of MDD and BD-I by the forced entry method** | | | | | |
| --- | --- | --- | --- | --- | --- |
|  | | | | | |
| Variable | Analysis by the forced entry method | | | | |
|  | B | S.E. | *p*-value | OR | 95% CI |
| Depressive temperament | –0.66 | 1.11 | 0.552 | 0.52 | 0.06–4.56 |
| Cyclothymic temperament | 3.75 | 0.96 | 0.000 | 42.63 | 6.53–278.57 |
| Hyperthymic temperament | 0.89 | 0.84 | 0.293 | 2.42 | 0.47–12.60 |
| Irritable temperament | –0.98 | 1.24 | 0.427 | 0.37 | 0.03–4.22 |
| Anxious temperament | –1.51 | 1.09 | 0.164 | 0.22 | 0.03–1.86 |
| PHQ-9 score | –0.01 | 0.03 | 0.699 | 0.99 | 0.94–1.04 |
| YMRS score | 0.12 | 0.07 | 0.071 | 1.13 | 0.99–1.28 |
| Constant | –3.03 | 1.76 | 0.086 | 0.05 |  |

Fit index of this model: χ^2^= 30.73 (*p*-value < 0.05), Cox-Snell R^2^ = 0.12, Hosmer–Lemeshow test *p* = 0.373, sensitivity = 0.21, specificity = 0.96, positive predictive value = 0.60, negative predictive value = 0.79, AUC of ROC = 0.72

dependent variable: diagnosis of MDD (1) and BD-I (2)

7 independent variables: scores of 5 subscales of the TEMPS-A and the severity of depressive and manic symptoms (PHQ-9 and YMRS scores, respectively)
