# Supplementary material for: Utility of TEMPS-A in differentiation between major depressive disorder, bipolar I disorder, and bipolar II disorder
Source: PLoS One. 2020 May 22;15(5):e0232459. doi: 10.1371/journal.pone.0232459 (PMC7244116; doi:10.1371/journal.pone.0232459)
Supplement: S2 Table — (DOCX) [file pone.0232459.s002.docx]

| **Table S2. Multivariate logistic regression analysis of the diagnosis of MDD and BD-II by the forced entry method** | | | | | |
| --- | --- | --- | --- | --- | --- |
|  | | | | | |
| Variable | Analysis by the forced entry method | | | | |
|  | B | S.E. | *p*-value | OR | 95%CI |
| Depressive temperament | –0.52 | 0.91 | 0.564 | 0.59 | 0.10–3.50 |
| Cyclothymic temperament | 3.00 | 0.80 | 0.000 | 20.09 | 4.15–97.24 |
| Hyperthymic temperament | –0.81 | 0.81 | 0.315 | 0.44 | 0.09–2.16 |
| Irritable temperament | 0.08 | 1.01 | 0.934 | 1.09 | 0.15–7.81 |
| Anxious temperament | –1.38 | 0.90 | 0.125 | 0.25 | 0.04–1.47 |
| PHQ-9 score | –0.01 | 0.02 | 0.580 | 0.99 | 0.94–1.03 |
| YMRS score | 0.10 | 0.06 | 0.067 | 1.11 | 0.99–1.24 |
| Constant | –0.86 | 1.45 | 0.550 | 0.42 |  |

Fit index of this model: χ^2^ = 26.63 (*p*-value < 0.05), Cox-Snell R^2^ = 0.09, Hosmer–Lemeshow test *p* = 0.830, sensitivity = 0.34, specificity = 0.88, positive predictive value = 0.63, negative predictive value = 0.68, AUC of ROC = 0.67

dependent variable: diagnosis of MDD (1) and BD-II (2)

7 independent variables: scores of 5 subscales of the TEMPS-A and the severity of depressive and manic symptoms (PHQ-9 and YMRS scores, respectively)
