# Supplementary material for: Utility of TEMPS-A in differentiation between major depressive disorder, bipolar I disorder, and bipolar II disorder
Source: PLoS One. 2020 May 22;15(5):e0232459. doi: 10.1371/journal.pone.0232459 (PMC7244116; doi:10.1371/journal.pone.0232459)
Supplement: S3 Table — (DOCX) [file pone.0232459.s003.docx]

| **Table S3. Multivariate logistic regression analysis of the diagnosis of BD-II and BD-I using the forced entry method** | | | | | |
| --- | --- | --- | --- | --- | --- |
|  | | | | | |
| Variable | Analysis using the forced entry method | | | | |
|  | B | S.E. | *p*-value | OR | 95%CI |
| Depressive temperament | –0.29 | 1.24 | 0.814 | 0.75 | 0.07–8.49 |
| Cyclothymic temperament | 1.13 | 0.94 | 0.227 | 3.11 | 0.49–19.57 |
| Hyperthymic temperament | 2.18 | 0.92 | 0.018 | 8.89 | 1.46–53.95 |
| Irritable temperament | –1.87 | 1.21 | 0.123 | 0.16 | 0.01–1.66 |
| Anxious temperament | –0.16 | 1.17 | 0.893 | 0.85 | 0.09–8.50 |
| PHQ-9 score | –0.01 | 0.03 | 0.783 | 0.99 | 0.93–1.05 |
| YMRS score | 0.03 | 0.06 | 0.630 | 1.03 | 0.91–1.17 |
| Constant | –1.98 | 1.70 | 0.243 | 0.14 |  |

Fit index of this model: χ^2^ = 9.94 (*p*-value = 0.192), Cox-Snell R^2^ = 0.06, Hosmer–Lemeshow test *p* = 0.008, sensitivity = 0.21, specificity = 0.94, positive predictive value = 0.63, negative predictive value = 0.70, AUC of ROC = 0.63

dependent variable: diagnosis of BD-II (1) and BD-I (2)

7 independent variables: scores of 5 subscales of the TEMPS-A and the severity of depressive and manic symptoms (PHQ-9 and YMRS scores, respectively)
