# Supplementary material for: Utility of TEMPS-A in differentiation between major depressive disorder, bipolar I disorder, and bipolar II disorder
Source: PLoS One. 2020 May 22;15(5):e0232459. doi: 10.1371/journal.pone.0232459 (PMC7244116; doi:10.1371/journal.pone.0232459)
Supplement: S4 Table — (DOCX) [file pone.0232459.s004.docx]

| **Table S4. Depressive symptoms assessed by the PHQ-9 and manic symptoms assessed by the YMRS in the subjects** | | | |
| --- | --- | --- | --- |
|  | MDD  (n = 176) | BD-II  (n = 112) | BD-I  (n = 58) |
| Severity of depressive symptoms |  |  |  |
| Remission (PHQ-9 score 0-4) : n (%) | 60 (34.1) | 31 (27.7) | 23 (39.7) |
| Mild depression (PHQ-9 score 5-9) : n (%) | 40 (22.7) | 26 (23.2) | 16 (27.6) |
| Moderate depression (PHQ-9 score 10-14) : n (%) | 37 (21.0) | 37 (33.0) | 2 (3.4) |
| Moderately severe depression (PHQ-9 score 15-19) : n (%) | 17 (9.7) | 9 (8.0) | 12 (20.7) |
| Severe depression (PHQ-9 score 20-27) : n (%) | 22 (12.5) | 9 (8.0) | 5 (8.6) |
| Severity of manic symptoms |  |  |  |
| YMRS score ≥ cut-off point of 8 : n (%) | 3 (1.7) | 5 (4.5) | 4 (6.9) |
